# Volunteer navigation partnerships: Piloting a compassionate community approach to early palliative care

**DOI:** 10.1186/s12904-017-0210-3

**Published:** 2017-07-03

**Authors:** Barbara Pesut, Wendy Duggleby, Grace Warner, Konrad Fassbender, Elisabeth Antifeau, Brenda Hooper, Madeleine Greig, Kelli Sullivan

**Affiliations:** 1 Faculty of Health and Social Development, University of British Columbia Okanagan, 1147 Research Road, Kelowna, BC V1V 1V7 Canada; 2 Faculty of Nursing, University of Alberta, 11405-87 Avenue, Edmonton, AB T6G 1C9 Canada; 3 School of Occupational Therapy, Dalhousie University, 5869 University Avenue, Halifax, NS B3H 4R2 Canada; 4 Faculty of Medicine and Dentistry, University of Alberta, 11560 University Avenue, Edmonton, AB T6G 2G2 Canada; 5 Interior Health Authority, Nelson, BC Canada; 6 Greater Trail Hospice Society, 1500 Columbia Ave, Suite 7, Rossland, BC V1R 1J9 Canada; 7 School of Nursing, University of British Columbia Okanagan, 1147 Research Road, Kelowna, BC V1V 1V7 Canada

**Keywords:** Hospice and palliative care, Volunteers, Compassionate community, Navigation, Public health

## Abstract

**Background:**

A compassionate community approach to palliative care provides important rationale for building community-based hospice volunteer capacity. In this project, we piloted one such capacity-building model in which volunteers and a nurse partnered to provide navigation support beginning in the early palliative phase for adults living in community. The goal was to improve quality of life by developing independence, engagement, and community connections.

**Methods:**

Volunteers received navigation training through a three-day workshop and then conducted in-home visits with clients living with advanced chronic illness over one year. A nurse navigator provided education and mentorship. Mixed method evaluation data was collected from clients, volunteer navigators, the nurse navigator, and other stakeholders.

**Results:**

Seven volunteers were partnered with 18 clients. Over the one-year pilot, the volunteer navigators conducted visits in home or by phone every two to three weeks. Volunteers were skilled and resourceful in building connections and facilitating engagement. Although it took time to learn the navigator role, volunteers felt well-prepared and found the role satisfying and meaningful. Clients and family rated the service as highly important to their care because of how the volunteer helped to make the difficult experiences of aging and advanced chronic illness more livable. Significant benefits cited by clients were making good decisions for both now and in the future; having a surrogate social safety net; supporting engagement with life; and ultimately, transforming the experience of living with illness. Overall the program was perceived to be well-designed by stakeholders and meeting an important need in the community. Sustainability, however, was a concern expressed by both clients and volunteers.

**Conclusions:**

Volunteers providing supportive navigation services during the early phase of palliative care is a feasible way to foster a compassionate community approach to care for an aging population. The program is now being implemented by hospice societies in diverse communities across Canada.

*“Everything comes at you so fast and there are so many decisions to make and you’re all sixes and sevens and when your volunteers come out we can sit and talk about this, and it helps us to understand a little more why they’re [healthcare] doing this or that. And oh, what a difference that makes.” (N-CARE Client)*

## Background

Volunteers play a vital role in palliative care through support, advocacy, and caregiver respite [1] in a variety of settings, including residential care, hospital, and home. The importance of their role is magnified in rural areas where healthcare resources are limited [2–4], and in the context of an aging population where early support [5], provided at home [6], is the optimal standard of care. Although research on volunteers is in a nascent stage, there is accumulating evidence of the benefits of volunteers for palliative patients and their families [1, 7, 8].

Developments in a public health approach to palliative care provide important rationale for the growth of volunteer capacity. Although the public health approach to palliative care has various understandings in the literature [9], the project reported in this paper builds upon the approach that recognizes the essential nature of social support to overall well-being [10], and the relevance of health promotion strategies for those on a palliative trajectory [11, 12]. This social support, public health approach requires strategic partnerships between governments, communities, and services to develop important social capital, which is characterized by relationships of trust, empathy, and cooperation. *Healthy communities* coordinate efforts to improve these essential partnerships; *compassionate communities* work to ensure that those most vulnerable benefit from broad-based support in accordance with their specialized needs [13].

Although the public health approach to palliative care is becoming more visible in the literature, there is yet little evidence to support this approach [14]. However, there are notable studies in progress. The INSPIRE study is evaluating the use of a volunteer-led social and practical support model for community dwelling adults in Ireland [15]. The ELSA study is examining the effect of a ‘social action’ volunteer on adult and informal carer outcomes in 12 sites in England [16]. One pilot study in Australia tested the impact of a “community network facilitator,” whose role was to mobilize social networks, on caregiver support. Caregivers participating in the intervention arm realized improvements on a number of outcome measures, however no significant differences between the control and intervention group were identified; a result that could be attributed to the small pilot sample size [17]. Results from these studies will contribute to our understanding of how a public health, compassionate community approach to care can impact outcomes for palliative clients and families in Western contexts.

Community-based hospice societies are strategically positioned to support the realization of a public health approach to palliative care. Indeed, recent evidence suggests that there is substantial scope for hospices to develop greater community engagement to assist those living with advanced life-limiting illnesses [18, 19]. However, a recent study exploring this public health approach in New Zealand indicated that although it was a priority for the majority of hospices studied, the means by which to realize that approach were less well-developed [20]. In this project, we sought to develop this compassionate community, public health approach through a model of volunteer navigation called N-CARE (Navigation: connecting, accessing, resourcing, engaging), designed to provide early palliative support in rural communities through community-based hospice societies.

N-CARE was developed based upon findings from a two-year trial of nurse navigation to support an early palliative population [21]. It is important to note that the public health approach to navigation described in this pilot is different from the health service approach to navigation, and hence, the volunteer roles differ. For example, cancer care has developed robust models of navigation that incorporate volunteers who assist individuals to navigate cancer services [22–24]. Comparably, the public health approach of the volunteer role in the N-CARE model was focused on improving older adult quality of life by assisting them to develop social capital and connections within their community. Moreover, the volunteer focused on meeting the quality of life concerns of the client through supportive interactions designed to inform, engage, and build belonging and security [13]. The navigation role also included identifying and addressing community gaps as part of a capacity-building focus. As such, N-CARE included key elements of a public health approach including the mobilization of community-based resources and supportive networks by trained volunteers who were overseen by a community-based organization and advisory committee. However, volunteers were trained and supported by an expert nurse navigator which facilitated some connections to formal healthcare services. In this paper we report the findings of the N-CARE pilot.

## Methods

**Fig. 1:**
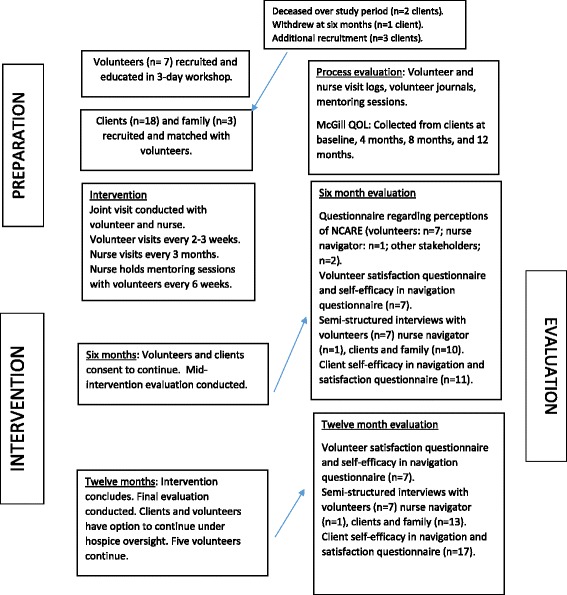
Study process

This pilot study was conducted in a single site conducted in a defined geographic area, consisting of three co-located rural communities, each with populations of 10,000 or less. The navigation intervention was piloted for 12 months (2015–2016). Mixed method evaluation was conducted at six months and 12 months using questionnaires and semi-structured interviews (see Fig. 1). Qualitative and quantitative data were collected concurrently, analyzed separately, and used to triangulate findings. Ethical approval was obtained from two universities and the health authority.

The intervention consisted of in-home visits by trained volunteers to provide navigation support. (see Fig. 1). Volunteers were supported by a nurse navigator who provided an oversight and mentoring role. Clients and volunteers were recruited as study participants. Client and volunteer screening, and obtaining informed consent, were done by the nurse navigator. To be eligible, clients had to be 55 years of age or older and have one or more advanced chronic illnesses that could reasonably lead to death within the next year (e.g., cancer, solid organ failure, neurodegenerative). The service was only available through the study, but clients could opt out of the evaluation and continue receiving the service if they desired to do so. Volunteers were required to have one year of volunteer experience (or equivalent background) and were screened through a criminal record check, reference checks, and an interview with the nurse navigator. After the volunteers received navigation training, the nurse navigator matched the client with a volunteer and a joint visit was conducted by the nurse and volunteer navigator. The volunteer navigator conducted independent visits according to the client preference (typically every two to three weeks) over 12 months. The nurse navigator conducted independent visits every three months to collect outcome data and to ensure the intervention was acceptable to clients. To facilitate the ongoing development of volunteers, the nurse navigator met with individual volunteers on an as-needed basis, as well as through group meetings that took place every six weeks. During these two-hour group meetings, volunteer navigators shared their experiences of navigation and provided mutual advice and support.

The study used a community-based research approach. A 14-member community advisory committee, with representation from hospice societies, medicine, pharmacy, nursing, municipal government, residential care, and the regional health authority, provided guidance to the project. Part of the role of this committee was to capitalize on the strategic partnerships that are essential to community capacity-building. For example, the connection to municipal government provided a forum through which to share existing community gaps.

A navigation curriculum, which included learning manuals, case studies, and workshop materials, was developed to prepare volunteer navigators. The curriculum addressed the following evidence-based [25] navigation competencies: understanding the navigator role, screening for quality of life concerns, advocating for clients, facilitating community connections, coordinating access to services and resources, and promoting active engagement. These six overarching competencies were further delineated into 27 specific indicators. Curriculum was piloted with the volunteers who participated in this study in April of 2015 during a three-day workshop. Minor revisions were made based upon feedback obtained from the workshop and from other key stakeholders including the community advisory committee, and provincial and national hospice and palliative care associations.

### Data collection and analysis

Testing the feasibility of data collection procedures was an aim of this pilot study. Volunteer navigators recorded the following data at each visit: duration and location of visit, people present, needs identified and addressed, and connections made. Volunteer navigators used a journal to document successes, challenges, and overall perceptions of the intervention. Group mentoring sessions conducted by the nurse navigator with volunteer navigators were audio-taped and transcribed. Transcripts of these sessions and volunteer journals were analyzed as part of the evaluation.

Volunteers completed self-efficacy and satisfaction questionnaires (see Figure 1). The volunteer self-efficacy in navigation questionnaire required responses to the stem “I feel confident in my ability to ,” using a 4-point Likert scale of strongly agree to strongly disagree, in relation to the 27 competencies upon which the navigation curriculum was constructed. The volunteer satisfaction questionnaire consisted of eleven questions (e.g., did you feel working as a navigator contributed to your satisfaction as a volunteer) to which volunteers were asked to respond using a 4-point Likert scale of strongly agree to strongly disagree. Clients completed self-efficacy, satisfaction, and quality of life questionnaires. The client self-efficacy questionnaire required responses to the stem “I have confidence in my ability to” using a 4-point Likert scale of strongly agree to strongly disagree, in relation to 4 questions (connection with healthcare system, connection with community resources, contacting volunteer, and communicating needs to others). The client satisfaction questionnaire consisted of 8 questions (e.g., were you able to complete the navigation activities you wanted to) to which clients were asked to respond using a 4-point Likert scale of strongly agree to strongly disagree. The McGill Quality of Life Questionnaire was used to measure quality of life [26, 27]; a key outcome goal for the navigation intervention. Questionnaire items were all worded positively (other than the McGill Quality of Life) and anonymised through the use of study identification numbers. Finally, clients/family, volunteer/nurse navigators, and other stakeholders completed a questionnaire about their perceptions of the N-CARE program and participated in semi-structured interviews, conducted by the Research Coordinator, about their experiences with N-CARE. Qualitative data were managed using NVIVO^QSR^. Thematic analysis was conducted using interpretive description [28]. Quantitative data were analyzed using SPSS^IBM^.

## Results

Seven volunteer navigators were recruited in the spring of 2015 through community hospice societies (See Table 1). Eighteen older adults and three family clients were recruited between April 2015 and February 2016 (See Table 2). Client recruitment was ongoing throughout the study to offset attrition due to death or study withdrawal. At the six-month evaluation, volunteer navigators and clients were invited to continue into the second six-month intervention period. All volunteers chose to continue. One client did not proceed into the second six-month period as he felt he no longer had need of the service. Two clients died while on the service.

**Table 1:** Volunteer demographics (*n* = 7)

Variable	Results
Age	Range: 53–70 Mean: 60.0
Gender	Male *n* = 0 Female *n* = 7
Length of time volunteering	>10 years: *n* = 3
	6–10 years: *n* = 1
	0–5 years: *n* = 3
Length of time volunteering with older adults	>10 years: *n* = 1
	6–10 years: *n* = 3
	0–5 years: *n* = 3
Volunteer agency experience	Hospice, Canadian Mental Health, Hospital, Residential Care, BC Cancer Society, Red Cross, Other

**Table 2:** Client demographics (*n* = 18)

Variable	Results
Age	Range: 56–85 Mean: 70.0
Gender	Male: *n* = 8 Female: *n* = 10
Living arrangements	Alone: *n* = 8 With Family: *n* = 10
Primary chronic condition	Cancer: *n* = 7 Other: *n* = 11

A description of the visit profiles is provided in Table 3. The intervention specified visits every two to three weeks, but volunteers were instructed to adapt this to the needs of the clients. Visits averaged approximately one hour in length. Volunteers were asked to document the nature of the services they provided. Table 4 provides examples, taken from the visit reports filled out by volunteers, of these services. Examples of navigation interventions included both individual (e.g., connecting individuals to community) and community capacity-building approaches (e.g., modifying community to support access). For example, one volunteer discovered that access to legal services was limited for those with mobility challenges and so advocated for changes in her community. One indication of the acceptability of an intervention is the number of cancelled visits. Over the one-year period there were 46 cancelled visits (15% of total booked visits), with the primary reasons being feeling unwell or having conflicting appointments. In total, volunteers dedicated 378.4 direct contact hours to visiting with older adults (calculated from visit duration recorded on visit reports). Volunteers devoted additional hours outside of direct contact time to explore potential solutions for clients. These hours were not recorded in this pilot. The cost of the nurse navigator over the one-year project was $37, 243 in wages. However, this cost included collecting and recording research-related data. Table 5 provides an overview of pilot evaluation findings.

**Table 3:** Visit profiles

Variable	Results
Total Participant (*n* = 18) Visits	Total Visits Reported: *n* = 252
	**Breakdown by provider:**
	Nurse Navigator: *n* = 59 visits
	Volunteer Navigators: *n* = 176 visits
	Combined Nurse and Volunteer Navigator: *n* = 17 visits
Days between visits	Mean: 17.88 SD:13.62
Number of visits received by individual participants	Range: 5–26 Mode: 17
Length of visits	In person: Range: 10–210 min Mean (SD): 86.24 min (39.45)
	By phone: Range 10–50 min Mean (SD): 20.23 min (11.28)
Declined visits	*N* = 46
	Reasons: Feeling unwell (*n* = 20); social/family obligations (*n* = 11); healthcare scheduling conflicts (*n* = 8); forgot visit (*n* = 3); other/unclear (*n* = 4)

**Table 4:** Examples of support provided by volunteers

N-CARE = Connecting, Accessing, Resourcing and Engaging
Connecting: Those things volunteers did to enable older adults to feel connected to others.
• Psychosocial support for “disappointments” inherent in the advanced illness trajectory. • Discussions about illness, coping, and overall life impact. • Social conversations. • Sharing of confidences difficult to discuss within family (e.g., discussions about death). • Family and neighbour mediation functions (e.g., helping to understand and resolve conflict). • Identification of friend and family connections and strategies on how to connect. • Visits while in hospital or residential care. • Strategies to reduce loneliness.
Accessing: Strategies that enabled clients to access the services and resources available.
• Assistance with reaching healthcare providers and making appointments. • Practical strategies to speak to healthcare providers about most pressing needs (e.g., reminder strategies, identifying problems, practice pronouncing physician names, notes to family to make physician appointments, how to bring up sensitive medical issues, how to understand physician’s behaviours and/or reluctance to act, use of advocates for appointments, advocacy to set up regular home physician visits, conversation plans). • Strategies to communicate wishes (e.g., care plan on refrigerator). • Mobility device options to support access. • Lawyers with wheel chair access. • Assistance with filling out forms (e.g., home owner grant). • Strategies to voice healthcare related concerns (e.g., letter writing). • Facilitating access to road tests prior to renewing drivers licence. • Discussions about ‘best’ choices (e.g., cost) in accessing resources such as transportation and help at home. • Flying options with airlines when accommodation required.
Resourcing: Identifying resources according to client need.
• Healthcare: physiotherapist, chiropractor, chronic illness self-help groups and services, alternative therapies, counseling. • Home support services: Meals on Wheels, housekeeping, free yard work, home delivery of oxygen. • Available living options in the community (e.g., assisted living, residential, rentals that accept pets). • Resources to assist with making life changes (e.g., low cost advertising for selling possessions, moving arrangements, places to donate treasured possessions). • Transportation and mobility (e.g., mobility aids, out of town travel assistance). • Identification of the best person to answer healthcare related questions. • Home safety/efficiency strategies (e.g., low cost kitchen appliance to replace broken one). • Comfort adaptations (e.g., therapeutic beds). • Personal safety strategies (e.g., replacing old shoes that could not be tied with supportive shoes with Velcro). • Advance care planning resources (e.g., options for organ donation). • Sources of special dietary needs. • Seniors resources (e.g., ombudsman, office of senior’s advocate, senior’s centre, adult day program). • Policy changes/services that affect seniors (e.g., information about changes to provincial health premiums for low income earners, palliative benefits).
Engaging: Strategies that assisted clients to engage more fully with life.
• Sounding board to assist clients with making decisions about their lives and transitions. • Options for self-management in relation to their experiences (e.g., implementing an exercise program to alleviate pain, watching educational videos about dialysis, relaxation exercises for sleep, strategies to monitor cognition). • Discussions about spiritual interests. • Renewing older hobbies or interests (e.g., coloring leading to art classes). • Seniors activity planning. • Playing games. • Advance care planning (e.g., funeral home visits). • Grief strategies to increase engagement with others after loss. • Design walking routes and an activity plan. • Facilitation of plan for philanthropic work (e.g., helping refugees moving into the community). • Strategies to keep pets. • Strategies for preparing for stressful events (e.g., renewing driver’s license). • Imagining options for living and thinking through quality of life issues. • Options of how to modify hobbies so that they are achievable (e.g., camping, berry picking). • Crafting an ‘emergency’ plan to deal with contingencies while caregiver is away.

**Table 5:** Overview of evaluation findings

Clients and Family	Volunteers
Confident in self-navigation as measured by self-efficacy questionnaire. Highly satisfied with intervention. Service rated as highly important to care because of: Assistance with making good decisions. Trusted, knowledgeable person available. Supported engagement with life. Increased awareness of available resources. Experiences put in context. **Areas for further development** More flexibility in visit schedules and types (e.g., in home versus telephone).	Well prepared in navigation as measured by self-efficacy questionnaire. Highly satisfied with role because of: Extended time for building relationship. Relationships of reciprocity. Positive perceptions of N-CARE program. **Areas for further development** More flexibility in visit schedule. Ensure sustainability of program. Additional education and mentorship. More awareness of program in community.

### Volunteer evaluation

Figure 1 provides an overview of the evaluation data collected from volunteers at six months and twelve months into the intervention. Results of the self-perceived confidence in navigation questionnaire indicated volunteers felt well-prepared. At the 12-month evaluation, means of all self-perceived competence items indicated satisfactory competence. Perceptions of the N-CARE program, gathered through the questionnaire and interviews, indicated that the program was well understood, well designed, and met a particular need in the community. Participants did indicate some concerns about the sustainability of the program in the community. Overall, volunteers indicated a high degree of satisfaction with the role on the satisfaction questionnaire. Volunteers indicated they would do the role again and would recommend it to others. Lower satisfaction was related to “Were you able to perform navigation services without difficulty?” and “Were you able to complete the navigation activities you wanted to?”

Qualitative interview data provided further insight into volunteer satisfaction. Volunteer navigators described the role as highly satisfying, largely because of the extended time for relationship-building. There was a strong sense of satisfaction in supporting clients over the long term. One participant described it as “*making it easier for them to go down that path (of illness)*.” Not only did volunteers feel like they were contributing to the lives of these clients, but they acknowledged how clients contributed to their lives. It was a relationship of reciprocity as expressed in the words of this volunteer. “*These people really touched me and I got a lot from them as well. I hope I gave them something but you know they became friends and part of my life.”*

Qualitative data also provided insights into why volunteers were less satisfied in their abilities to perform the navigation role and activities. It took time for volunteers to develop an understanding of their role. During the first six months they described feeling like they should be doing more for clients as opposed to spending time building relationship. Throughout the intervention period it was typical for the volunteers to question the value of their contributions. And if they were not clear on the role, it was challenging for them to construct criteria by which to measure how well they were performing in that role. In pondering this dilemma one volunteer suggested “*sometimes just providing that safe environment for someone to ventilate about their issues – maybe that’s my criteria*.” However, as they spent more time with clients they began to understand that the role was not limited to navigating resources but that it was also about ‘*helping people navigate this time of their life on an emotional level.*”

Volunteers expressed several recommendations for program development during the interviews. They suggested that the visit schedule should have even more flexibility. Clients had fluctuating needs and varied social support and so it was important to take that into account in a person-centered approach. They further spoke of the importance of the mentorship provided by the nurse navigator, suggesting that this mentorship could be expanded even further. As they learned the scope and boundaries of this new role, it was essential that they have someone knowledgeable and supportive to whom they could turn.

When responding to the questionnaire about their perceptions of the N-CARE program, volunteers reported a high degree of support for the program in their community. They were satisfied with how it was planned and implemented; however, they were less certain about how it could be sustained beyond the research period, largely because of the need to recruit early palliative clients. Of particular concern were the resources required to enhance public awareness of the program; although, volunteers expressed a number of innovative ideas on how to increase public awareness.

### Client and family evaluation

Clients and family participated in an evaluation at six months and twelve months. Evaluation conducted at six months focused on client and family understandings of navigation, experiences of helpful interventions, challenges encountered, and perceived importance of the service. Clients and family were highly satisfied. When asked during the interviews how important the service was to them, the mean response was 8.6/10. Client scores on the self-efficacy in navigation questionnaire ranged from 2.91–3.55 on a 4-point rating scale with higher scores indicating better confidence. The collection of quality of life data using the McGill Quality of Life Questionnaire was feasible and acceptable to clients. Clients and family who participated in interviews at six months demonstrated an overall understanding of the role of the volunteer navigator (e.g., support); although, the idea of “navigation” was not well understood. Benefits of having a volunteer navigator included confidence in being able to ask for help, having someone knowledgeable and available, knowing there was backup when needed, helping put experiences into context, and bringing awareness of available resources. Challenges described by clients included focussing too much on the client’s illness, providing too much information, and trying to find time for volunteer visits amidst other medical appointments.

Evaluation conducted at 12-months indicated that all clients agreed or strongly agreed with statements on questionnaires regarding their confidence in self-navigation and their satisfaction with N-CARE. The only exception was two clients who disagreed with items related to being connected to the healthcare system and one client disagreed with being connected to community resources. The 12-month interview, conducted at study conclusion, focused on reasons for enrolling with the program and overall experiences and satisfaction with the service. Themes constructed from the data were clients’ experiences that made the service important, experiences of the intervention itself, and benefits of the intervention.

#### Experiences that made the N-CARE service important

Participants spoke eloquently about the experiences of having a chronic illness that led them to participate in the N-CARE project. Having a complex, advanced chronic illness led to feelings of stigma, isolation, loneliness, and disappearance of self. For example, this participant spoke of the experiences of developing cancer. “*It causes a feeling of isolation and aloneness. Others treat you differently.”* Coping with illness over an extended period of time could have devastating effects on self. As one participant shared “*when you’ve been sick for a long time [voice breaking], after a while everything disappears, and all your stability [crying].”* These effects were heightened if both partners where coping with a complex chronic illness. One participant described it as “*two illnesses fighting here, you know, there’s two sick people. And once in a while you say things that you’d never say normally and it takes you back again and again. Is this the person I have become? You know it’s not a nice way to feel.”*

This experience of advanced chronic illness was compounded by the challenges of aging. Participants spoke of the disrespect that accompanies aging in our society, and the resulting feelings of being patronized, which ultimately led to feelings of being “in the way.” One couple shared of the dilemma of trying to stay independent amidst increasing needs. They wanted to reach out for help but were not sure that their needs justified the time of the navigators. “*I hope you are not wasting your time with us. But we knew they [navigators] weren’t and I guess we wanted them to say no [you are not wasting our time]*.” Another participant expressed surprise at finding herself elderly and wondering what services she should be looking for as an “elderly” person. “*I have never been sick my whole life and so this being older is a new experience for me, so let’s see what it’s all about.*”

The theme of not wanting to be a burden to others, or to the healthcare system, was prevalent. Participants were aware of the media attention being given to the healthcare costs of an aging population. They also did not want to be a burden to family and friends as a result of their increasing psychological needs. Moreover, seeing professionals such as a psychologist for what participants considered normal aging events could be perceived as making them “*feel weaker.”*

A primary reason for registering with the service were challenges with the healthcare system which was perceived by clients as cold and fragmented. Those with cancer struggled with fragmentation between the primary care system and the cancer care system. Although participants spoke highly of their primary care physicians, they felt physicians simply did not have time to help them solve their complex challenges or signpost common disease trajectories. One participant described having a number of psychological needs related to being a caregiver while struggling with her own complex illness. *“My doctor, I feel doesn’t know me enough to understand and she doesn’t have the time to put into that. So that’s where your service comes in.”*

#### Experiences of the intervention

Participants commented on the characteristics of the volunteers and the nature of the navigation visits. Participants valued volunteers who approached them professionally, but with a lay attitude. Volunteers were described as good listeners, caring, personable, outgoing, friendly, patient, positive, capable, conscientious, kind, non-intrusive, and diligent in finding out what they did not know. Visits with volunteers were described as non-intimidating, warm, welcoming, and respectful. The timing of visits was not always optimal. Participants recommended that the visit schedule be more flexible. For example, they suggested that six week intervals with the option for telephone calls in between might be more appropriate during times of stability.

The focus and nature of the volunteer navigator visits were not clear to clients at the outset, primarily because they were confused by the concept of navigation. Some were disappointed there was not more instrumental support (e.g., providing transportation or housecleaning). However, as their relationships with volunteers developed, participants described the importance of the visits in meeting their needs. “*It was, ‘how are things going’ and then I opened up my mouth and we started to talk about what I felt we needed to talk about. It didn’t seem like they were on this or that, or we must talk about this today. It was more fitting of my needs each time.*” Participants had a need to talk about their chronic illness and so appreciated that volunteers had some healthcare background; however, they did not expect healthcare advice. As one participant said, ‘*It’s not so much about the medical; it was putting things in context*.”

#### Benefits of the intervention

Older adults and family described four primary benefits of being part of the N-CARE service: making good decisions for both now and the future, having a surrogate safety net, supporting engagement with life, and, hence, transforming the nature of their illness experience. Volunteers performed roles of helping clients identify their needs and making good decisions around meeting those needs. Older adults referred to this as helping them with a *“sober, second opinion,” “getting their house in order*” or “*getting them out of the woods*.” Volunteers helped clients weigh options related to housing, finances, treatment decisions, transportation, and advance care planning. Volunteers helped participants sign-post, or identify what might be coming down the road, so they could make good decisions in light of what they had identified. One family spoke of feeling overwhelmed with options and questions, suggesting the timely information provided by the volunteer would be important in the future for her caregiving role. “*What will help me as the caregiver is that I will be able to phone them [volunteer] and they’ll be able to put me where I am at, and what I should be doing, and that will be more than helpful*.”

The volunteers also provided a surrogate “*safety net*.” Two participants were experiencing gaps in formal health services because of rural shortages and so spoke of the role the volunteer played in supporting them during this time. Without the psychological safety provided by the volunteer, clients spoke of feeling anxious about the future. For example, one couple who were both coping with chronic illness, and had no local family, valued the fact that they could call upon the volunteer if at some point they reached a “*crisis point.”* Volunteers also provided a measure of safety around what could be discussed. They were described as “*friends but not quite friends*” and their capacity to engage with deeper issues that family members felt uncomfortable talking about was important. For example, one participant shared that he and his spouse would never have entered the kinds of conversations they did without the presence of the volunteer. Another participant said “*we have conversations that are, like I say, going deeper into cancer, and that’s a useful conversation for me to have.”* Safety was also found in the advocacy role of volunteers; “*I cannot think of the word right now…somebody that defends you. So she was good in clearing that kind of path for me. And taking away some anxiety.”*

Engagement was another benefit cited by clients. Participants appreciated the ways in which volunteers supported their capacities. *“I was surprised by how much it does help the minute she comes in. And later on I said to someone just the other day, ‘You know I did this and I didn’t realize I could still do it.’”* One participant spoke of how she vacillated between wanting to be dead or alive and how the engagement she experienced through the program helped her. “*Somehow you feel more alive when you’re ahead of the game…you’ve got something going there…this program does help.”* Another participant suggested that the service kept him from slipping into depression after a series of losses and how he now felt productive and connected once again.

Overall, participants suggested that volunteers transformed their illness experience. *“It showed us that other people have got the same problems…it gives us an uplifting…I’m sure other people have the same feelings we have…if someone’s there just to put a hand on your shoulder we know we’re going through it with other people.”* Knowing there was a program for their unique needs helped to offset feelings of loneliness and neglect. “*Somebody is looking out for the seniors [through N-CARE] and trying to find outside of their own little world of experience what is happening with the rest of us.”* One couple spoke of how easy it was to lose pride in existence as you get older and how “*life problems gang up on you.*” The attention they received from the volunteer helped to re-instate that pride. One participant described the volunteer as a placebo. “*Cancer is not just the physical thing, it’s an emotional wound. She’s sort of like a placebo in that she’s not going to cure my cancer, but she makes it a lot more livable*.” Participants further spoke of humorous and uplifting moments they enjoyed with the volunteer. In this way participants were acknowledging how the intervention altered the qualitative nature of their experience, even if it did not change the medical trajectory.

## Discussion

The results of this pilot are promising. Recruitment was successful with little study attrition. Recruitment was facilitated by the community-based nature of the research whereby community members were involved with study conceptualization and implementation. We attribute the lack of attrition, other than through death, to the year-long nature of the pilot in which volunteers and clients had extended time to build trust and relationship. The majority of visits occurred as mutually planned, with most cancellations occurring because of poor health. Volunteers demonstrated a high degree of resourcefulness in their navigation activities, found the role satisfying and meaningful, and would recommend it to others. After some initial role confusion, volunteers and clients partnered to meet a wide variety of needs through connecting to others, finding resources, facilitating access to those resources, and engaging clients with things that supported their quality of life and independence. Clients described a transformation of their illness experience that was similar to what was reported in a systematic review of public health interventions at end of life [14]. A key theme from that review was how these interventions made a practical difference that transformed the immediate experience of illness.

Assistance with decision-making was one of the most important benefits cited by clients. For example, volunteers facilitated conversations about meaning, and hence, contributed to clients’ sense of control which assisted their decision-making. This is similar to other research where this sense of control has been shown to be important for an early palliative population [29] and where volunteer interventions that focused on communication, emotional support, education, and advocacy targeted the priority needs [19]. Navigation activities performed by volunteer participants extended beyond building individual capacity to building community capacity, again a key theme from the review cited above [14]. They advocated at a community level for the resources required by their clients. As a result of this prolonged and intensive involvement with a volunteer, clients and families described a milieu of support that facilitated a sense of belonging, safety, and engagement. This community-based development of social capital exemplifies a public health, compassionate communities approach to care for this population [13].

A number of lessons were learned that will be applied to future offerings of this program. First, with a new role such as this, it will be important to provide ongoing education and mentoring. The nurse navigator on this project provided this ongoing mentorship. Although she provided little direct support to clients, her role in supporting volunteers was important and could be expanded further to include structured ongoing education. Previous research has suggested that the priority needs for volunteers working with this population are emotional support, role clarification, and continuing education [19]. These priority needs can be met concurrently through sessions that provide both structured education and unstructured opportunities to connect with other volunteer navigators for emotional support and role development. Second, the program will be designed around more flexible in-home visit schedules with telephone contact between home visits. However, regular visits in the early phase of volunteer/client relationship will be encouraged so that relationship and rapport can be established. This is particularly important in light of the six month evaluation data suggesting that clients and volunteers were still in the formative stages of understanding their partnership in the context of navigation. Third, more focused communication strategies will be implemented to raise community awareness of the program. This will be particularly important to ensure that appropriate clients are identified and made aware of the services available to them.

Although the pilot results were promising, there are two important limitations. First, this program was implemented within a single geographic region in which the research team had been involved for many years. A previous project in the community had piloted a nurse navigator and so the community was well-prepared for this next step. With this expanded scope for the volunteer, this previous research and community partnership was essential to ensuring that the program was implemented in a way that was acceptable to all stakeholders. Second, although the volunteers were mentored by a nurse navigator, N-CARE was only loosely connected to formal healthcare services. The nurse navigator had been nursing long-term in this geographic region and so had intimate knowledge of the persons and services available, although she was not employed by the health region during this project. This lack of a stronger connection with healthcare may present difficulties in the future for recruiting clients and for connecting clients with needed services. Ideally, a program of this nature will work in partnership with primary care. There is a need in future to determine how to most effectively link these volunteers more strategically to existing in-service healthcare partners. Effective linking will ensure that volunteers get the referrals and support they require to do their role while maintaining the community-based, public health nature of the intervention.

## Conclusion

A program such as N-CARE has the potential to meet three important needs: early support to improve the quality of life of older adults living with advancing chronic illness, a satisfying and meaningful role for volunteers, and a way to support a compassionate communities approach to palliative care. N-CARE is currently being implemented and evaluated in diverse rural and urban communities across Canada. The objectives of this scale up of N-CARE are to describe the adaptations that support best-practices for implementation in diverse contexts; to evaluate outcomes at the individual, organization, and community level; and to plan for sustainability. Future studies are required to explore outcomes of the N-CARE program in relation to standard care.

## Abbreviations

N-CARE: Navigation: connecting, accessing, resourcing, engaging
